# Prevalence and risk factors of intestinal parasitic infestations among preschool children in Sekota town, Waghimra zone, Ethiopia

**DOI:** 10.1186/s12887-019-1774-2

**Published:** 2019-11-14

**Authors:** Mesfin Wudu Kassaw, Ayele Mamo Abebe, Kenean Getaneh Tlaye, Alemu Birara Zemariam, Biruk Beletew Abate

**Affiliations:** 1 Department of Nursing, College of Health Science, Woldia University, Po Box 400, Woldia, Ethiopia; 2 Department of Nursing, College of Health Science, Debre Berhan University, Po Box 400, Debre Berhan, Ethiopia

**Keywords:** Intestinal parasites, Preschool children, Sekota town, Prevalence, Risk factors, Infestations

## Abstract

**Background:**

Intestinal parasitic infestations triggered considerable gastrointestinal morbidity, malnutrition, and mortality worldwide. In particular, young children in developing countries affected most. Helminthiasis infestation accounts for 10–20% of prevalence on preschool children worldwide. Unfortunately, small children below 5 years are uniquely susceptible to intestinal parasitic infestations in poor communities. This is because of children’s behavior like playing with soil and putting hand -to- mouth habit. Thus, the aim of this study was to assess the prevalence and risk factors of intestinal parasitic infestations among preschool children in Sekota town, Ethiopia.

**Methods:**

A community-based cross-sectional study was carried out on 378 preschool children in Sekota town from February 15 – March 10/2019. Stool specimens were collected and examined for intestinal parasites using wet mount and formal ether concentration technique. The risk factors of intestinal parasites were assessed using a pretested structured questionnaire. The data were entered and analyzed using Epi-data version 4.2.0.0 and SPSS-version 23 statistical software respectively. Both bivariable and multivariable analysis was carried out, and potential co-linearity was tested for closely similar variables. Variables with *P* value less than 0.05 in multivariable analysis was considered as statistically significant and reported with 95% CI and odds ratio.

**Results:**

The prevalence of intestinal parasitic infestations in Sekota town on wet mount and formal ether concentration techniques was 83(21.9%), (95% CI, 17.7–26.3%) and 113(29.9%), (95% CI, 25.1–34.8%) respectively.

In multivariable analysis, not taking medication as periodical deworming (AOR, 95% CI), (2.5, 1.5–4.3), presence of animals in the living room (AOR, 95% CI) (3.1, 1.8–5.3), and being a government employee as an occupation (AOR, 95% CI), (3.4, 1.1–10.0) were increasing the odds of intestinal parasitic infestations.

**Conclusions:**

The prevalence of intestinal parasitic infestations in Sekota town is high, which is a public health problem. The risk factors that contributed to intestinal parasitic infestations in this study were preventable and modifiable. Therefore, the concerned bodies need to emphasis on periodical deworming and keeping animals in separate room.

## Background

Healthy children are a vital resource to ensure the future wellbeing of a community. Regardless of these, quality of family living conditions, prevalence, and mode of transmission of infectious diseases and nutrition are among the strongest immediate determinants that cause morbidity and mortality in children younger than 5 years of age [1]. Of these, intestinal parasites are the major problems of children, which colonize the gastrointestinal tract and manifested as diarrhea, vomiting and abdominal cramp [2].

The parasitic infestations are acquired by ingestion, and or penetration of the skin through the infective forms of the parasites [3]. The infestations were higher on children raised with mothers, who have poor hygienic practices. Poverty, illiteracy, poor hygiene and lack of access to portable water were indicated as risk factors of intestinal parasitic infestations. A warm and humid tropical climate were also risked factors for intestinal parasitic infestations in tropical and sub-tropical countries [4].

Intestinal parasitic worm infestations are distributed virtually throughout the world and are still a serious public health problem in the world. Developing countries affected most with a variety of health hazards [1]. In estimation, intestinal parasites affect approximately 3.5 billion persons worldwide. Of these 450 million people develop clinical morbidity. Many of these are children from developing countries. Of the affected people, 1.47 billion were infected with *roundworm*, 1.3 billion were infected with *hookworm* and 1.05 billion were infected with *whipworm* [3].

Preschool children were attributable to the prevalence between 10 and 20% of the two billion people with helminthiasis worldwide. Regarding the types of worm infestation, 21 million preschool children were infested with *Hookworm,* 122 million were infected with *Ascaris lumbricoides* and 86 million were infested with *T. trichiura* [5]. Intestinal parasitic infestations are endemic worldwide and have been described as constituting the greatest single worldwide cause of illness and disease [6].

Every year 1400 million children were infected globally with a worm infestation. Epidemiological surveys have been revealed poor sanitation and inappropriate environmental conditions coupled with indiscriminate defecation, geophagy and the contamination of water bodies as the most important predisposing factors of intestinal worm infestation [7].

The prevalence and intensity of intestinal infestations were high in developing countries, particularly among populations with poor environmental sanitations [8].

In Ethiopia, intestinal parasitic infestations were widely spread [9]. The distribution and prevalence of various species of intestinal parasites differ from region to region because of several environmental, social and geographical factors [10, 11].

Small children below 5 years were uniquely prone to intestinal parasitic infestations in a rural community. This is because of childhood behavior. They play in the mud, suck their fingernail, and eat soil. They have no habit of handwashing before a meal and after touching dirty things and have less toilet hygienic training. When these combined with low socioeconomic status, and low educational status of parents, mainly mothers, the transmission, and distribution of the infestations reach the peak [12, 13].

Parasitic infestations cause serious public health consequences such as iron deficiency anemia, growth retardation, physical and mental health problems, loss of weight in pregnancy and low birth weight [14].

The interventions of intestinal parasitic infestations emphasis on control and prevention strategies including regular anti-helminthic treatment and improved water supply, sanitation, and health education [15, 16].

Health related Epidemics Were a major health hazard of Sekota town because of low levelenvironmental and individual hygiene as well as poor preventive public health services. Only 32.2% of Sekota town’s population had access to clean water. Seventy-two percent of all urban residents had access to portable water and 11.7% of the households had access to protected well and spring water [17].

Most of the previous studies conducted in Ethiopia had focused on school-age children, even limited studies had been reported the prevalence of intestinal parasitic infestations among under-five children [9, 11, 16, 18]. This means, the data about intestinal parasites on preschool children in Ethiopia has scarcity. Therefore, this study is designed to assess the prevalence and risk factors of intestinal parasitic infestations among preschool children in Sekota Town, Wag Himra zone, Ethiopia.

## Methods

### Study design and population

A community-based cross-sectional study was carried out on 378 mother-preschool child pairs in Sekota town from February 15 – March 10/2019. The town has two kebeles, and both of the kebeles were included in the study. Mothers and their children, who completed their second years of birthday and less than the birthday of year six were the study population from both kebeles.

**Eligibility criteria:** mothers with their children, aged 2 to 6 years and lives in Sekota town at least for 6 months were included. Mother-child pairs whose children took standard treatment for intestinal parasites in the last 6 months and mothers who have seriously ill children were excluded.

### Operational definitions

**Preschool children:** children whose age is between the first day of year 2 and 6 years but not attending their 6 years of birthday**.**

**Prevalence:** the total number of intestinal parasitic cases per 100 of the total samples identified on formal ether concentration technique.

**Intestinal parasites**: are parasites that can infest gastrointestinal tracts of the human body.

**Parasitic infestations**: intestinal parasite infestation is getting positive laboratory result for ova or any parasite stage and confirmed by stool examination.

**Fingernail cleanness –** the absence of dirt or dust under the nail of the child was considered as clean and the presence of dirt or dust under the nail considered as unclean fingernails on observation.

### Sample size determination

The sample size, 384 was determined using a single population proportion formula. The population proportion used for calculation from the previous study was (*p* = 52.3%) [19]. While calculating the sample, a 10% non-response rate, 95% CI, and 5% margin of error were considered, where n is the required sample size.

### Sampling procedures

Sekota town has two kebeles and the study participants were sampled from both kebeles using simple random sampling technique. The participants, mother -child pairs were found using under-five children registration book available at kebele office. The sample size was proportionally allocated to each kebele based on the number of preschool children. The study participants were selected from a list of preschool children, kebele registration book.

**Fig. 1:**
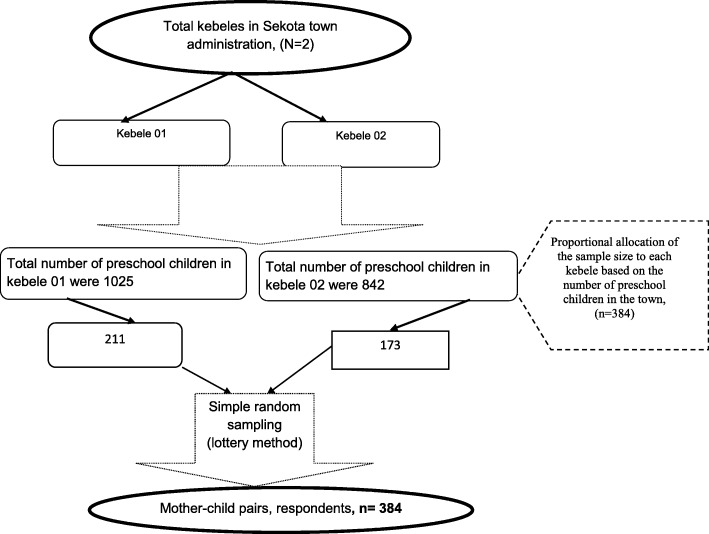
Schematic diagram of the sampling procedure for the study, “prevalence and risk factors of intestinal parasitic infestations among preschool children in Sekota town, Waghimra zone, Ethiopia, 2019

From the registry book, children whose age was 2 to 6 years old house’s number was selected. When two and above children registered from one house, only the house number was taken once and one child was selected randomly by lottery method. A total of 384 preschool children were supposed to be included in the study. Mothers of children whose children’s stool sample collected were also contacted for an interview. When the child’s mother cannot be reached, the immediate caregiver was interviewed (Fig. 1).

### Data collection tools

The interview part mainly the socio-demographic data was collected from mothers by four senior bachelor nurses who were experienced and took 1 day training using structured interview questioners. Mothers of a selected child were requested for their willingness to provide the stool samples from their children on a small piece of plastic sheet as well as for their involvement in the interview.

### Stool sample collection and examination

The mothers were adequately instructed on how to get a little portion of their children’s stool into the plastic sheet. Fresh stool samples were collected by mothers from their children. A clean piece of labelled plastic sheet along with applicator sticks was distributed to each selected mother -child pairs. The samples were received from mothers by one bachelor of laboratory technologist. The date of stool sampling, the identification number of the participant, age and sex was recorded for all subjects on a recording format. The microscopic examination, the transfer, and the formal ether concentration techniques were done by the other three laboratory technologists independently and blindly.

**Direct microscopic examination**; a direct microscopy examination was performed at the site of stool sample collection, Sekota health center immediately for possible detection of various parasite’s stages of development without preservation. All developmental stages of the parasites (cyst, egg, larvae and adult) were recorded. The direct microscopy or wet mount examination was prepared by emulsifying the stool samples using a drop of normal saline (0.85% NaCI) on a slide. After microscopic examination an approximate of pea-size of stool samples were preserved in 10% formalin containing tube before transported to Sekota Hospital laboratory for formal ether concentration technique.

**Formal-ether concentration technique**; The stool samples were emulsified using 4 ml of 10% formol water suspension which was strained to remove large fecal particles. Then 4 ml of ether was added and the tube mixed for 1 min and immediately centrifuged at 750–1000 g (3000 revolution per minute for 1 min). After centrifuging, the parasites sedimented to the bottom of the tube and the fecal debris collected in a layer between the ether and formol water. After discarding the supernatant, the sediment was transferred to a slide and covered with a cover glass. The sediment was examined microscopically for cysts, oocysts, eggs and larvae of intestinal parasites at Sekota Hospital. The portion of stool samples processed and examined microscopically using both direct wet-mount and formal-ether concentration techniques were following the WHO procedures or guidelines [20, 21].

### Data analysis

The data was entered to Epi-data version 4.2.0.0 and analyzed using SPSS version 23. The descriptive statistics were expressed as percentages and frequencies. The association between independent variables and the dependent variable were computed. Variables found to have an association at *P* value < 0.25 in bivariable with both COR, and 95% CI consideration entered to multivariable logistic regression to test for independent association and to control confounding variables. Potential co-linearity was considered and tested. Variables with *P*-value less than 0.05 in multivariable analysis were measured as statistically significant with 95% CI and AOR.

### Data quality control

For each step standard operational procedures (SOP) were followed. The socio-demographic questionnaire was pre-tested on 5% (20 mothers) of the samples in Woldia town. The interview guide was edited accordingly for virtual data collection. Data was checked for its completeness, and missing information at each point by all investigators and data collectors. Data collectors, and laboratory technologists were taking a 1-day training in two separate rooms. The laboratory technologist’s training was supported by laboratory practice at Woldia university, college of health science, department of laboratory, laboratory room. At the end of data collection and stool sample examination, a recheck was made on 10% of stool samples by senior and experienced laboratory technologists at Woldia University, laboratory room. Those personnel did not have information about the previous result.

The recheck confirm the absence of discrepancy with the primary data. One microscope and centrifuge were checked and labeled by senior laboratory technologist to be used for examining the parasites by discriminating from other microscopes and centrifuges. All the stool sample collectors, microscopic examiners, sample transferrers, and formyl ether concentration examiners were blinded up to the end of the research and later as a whole including the recheck of the sample.

## Results

### Children sociodemographic status

The mean age of children was 4.24 years with a standard deviation of 1.02 years (x ***±*** sd), (4.24 ***±*** 1.025). A total of 378 mother-child pairs were involved in this study and yields a 98.4% response rate. Regarding the sex of preschool children, 190 (50.3%) were males and 156 (41.3%) of the children were completed vaccination. More than half of the children, 206 (54.5%) were had history of diarrhea. Almost all of the children 370 (97.9%) had no history of diagnosed chronic medical cases, mainly diabetic millets, renal failure, heart failure, developmental retardation and epilepsy on maternal complain only (Table 1).

**Table 1:** Sociodemographic, behavioral, and medical history of preschool children in Sekota town, Waghimra zone, Ethiopia, 2019, (total positive for IP, *n* = 113), and total negative for IP, *n* = 265)

Variables	Categories	Parasitic infestations		Frequency	Percent
		Negative No. (%)	Positive No. (%)		
Sex of children	Male	127 (66.8)	63 (33.2)	190	50.3
	Female	138 (73.4)	50 (26.6)	188	49.7
Frequency of child illness in the last 1 year	1 Times	108 (69.7)	47 (30.3)	155	41.0
	2–3 times	79 (83.2)	16 (16.8)	95	25.1
	**>** 3 Times	78 (60.9)	50 (39.1)	128	33.9
Habit of playing with soil	Yes	227 (68.4)	105 (31.6)	332	87.8
	No	38 (82.6)	8 (17.4)	46	12.2
Habit of waking in bare foot	Yes	190 (69.3)	84 (30.7)	274	72.5
	No	75 (72.1)	29 (27.9)	104	27.5
History of deworming intake	Yes	122 (78.2)	34 (21.8)	156	41.3
	No	143 (64.4)	79 (35.6)	222	58.7
Vaccination completion	Yes	234 (72.0)	91 (28.0)	325	86.0
	No	31 (58.5)	22 (41.5)	53	14.0
Enrolment in school	Yes	103 (67.3)	50 (32.7)	153	40.5
	No	162 (72.0)	63 (28.0)	225	59.5
Habit of child shoes wearing	No wear at all	8 (29.6)	19 (70.4)	27	7.1
	Sometimes	197 (73.0)	73 (27.0)	270	71.4
	Always	60 (74.1)	21 (25.9)	81	21.4
Habit of outdoor spent	Yes	219 (72)	85 (28)	304	80.4
	No	46 (62.2)	28 (37.8)	74	19.6
Diagnosed medical cases	Yes	4 (50%)	4 (50%)	8	2.1
	No	261 (70.5)	109 (29.5)	370	97.9
History of diarrhea	Yes	143 (69.4)	63 (30.6)	206	54.5
	No	122 (70.9)	50 (29.1)	172	45.5
Fingernail cleanness	Yes	86 (71.7)	34 (28.3)	120	31.7
	No	179 (69.4)	79 (30.6)	258	68.3

Key; IP-intestinal parasites, and No. = Number

### Environmental characteristics

In 235 (62.2%) of households, there was latrine but majority of the remaining households used open field excreta disposal options, near the home, 82 (57.3%), and in the farm/river 44 (30.8%). In addition, 201 (53.2%) of the households had animals in the same living room.

### Prevalence of intestinal parasitic infestations

**Fig. 2:**
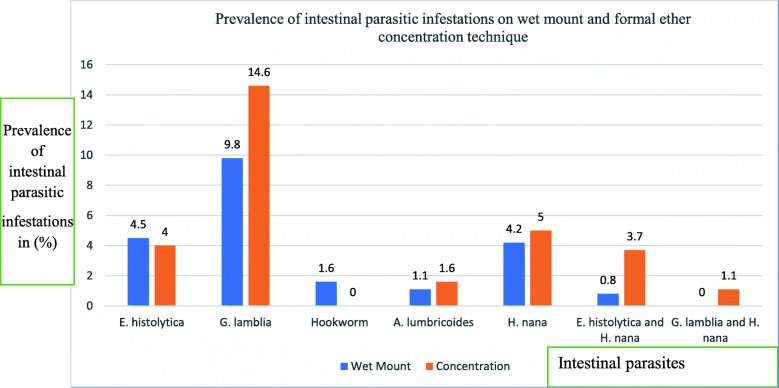
Prevalence of intestinal parasitic infestations on wet mount, and formal ether concentration technique in Sekota town, Waghimra zone, Ethiopia, 2019

The prevalence of intestinal parasitic infestations in Sekota town was determined using both wet mount and formal ether concentration techniques. The prevalence was 83 (21.9%) with 95% CI of (17.7–26.3%), and 113 (29.9%) with 95% CI of (25.1–34.8%) on wet mount and concentration techniques respectively. In the study area *Entamoeba histolytica\dyspar cyst, Giardia lamblia cyst, Ascaris lumbricoides, Hookworm,* and *Hymenolepis nana* were examined. There was also a co -infestation of *Entamoeba histolytica\dyspar cyst* and *Hymenolepis nana* as well as *Giardia lamblia cyst* and *Hymenolepis nana* (Fig. 2).

### Risk factors of intestinal parasitic infestations

All variables which had a *p*-value less than 0.25 in bivariable analysis were transferred to multivariable logistic regression analysis. In this study all variables were included for multivariable analysis, because those variables which had *P* value > 0.25 were the interests of the authors. On multivariable analysis not taking medication for deworming (AOR, (95%CI), (2.515(1.482–4.269), presence of animals in the same living room (AOR, (95%CI) (3.104(1.829–5.267), and being a government employee as an occupation (AOR, (95%CI) (3.365 (1.132–10.005) were increasing the odds of intestinal parasitic infestations (Table 2).

**Table 2:** A multivariable logistic regression analysis of potential risk factors with parasitic infestations identified using formal ether concentration technique among preschool children in Sekota town, Waghimra zone, Ethiopia, 2019, (total positive for IP, *n* = 113), and total negative for IP, *n* = 265)

Variables	Categories	Parasitic infestations		COR	*P*-value	AOR	95% CI	
		Negative (%)	Positive (%)					
							Lower	Upper
History of deworming intake	No	143 (64.4)	79 (35.6)	1.99	0.001	2.51	1.48	4.27
	Yes	122 (78.2)	34 (21.8)	1		1		
Complete Vaccination	No	31 (58.5)	22 (41.5)	0.55	0.19	0.64	0.33	1.25
	Yes	234 (72.0)	91 (28.0)	1		1		
Habit of playing with soil	Yes	227 (68.4)	105 (31.6)	2.20	0.59	1.23	0.58	2.59
	No	38 (82.6)	8 (17.4)	1		1		
Habit of shoes wearing	Regularly	60 (74.1)	21 (25.9)	1		1		
	Sometimes	197 (73.0)	73 (27.0)	1.06	0.79	1.09	0.59	1.99
	Not wear	8 (29.6)	19 (70.4)	6.79	0.39	0.60	0.19	1.89
History of diarrhea	No	122 (70.9)	50 (29.1)	1.08	0.93	1.02	0.63	1.67
	Yes	143 (69.4)	63 (30.6)	1		1		
Occupation	Merchant	36 (83.7)	7 (16.3)	1		1		
	Housewife	188 (68.4)	87 (31.6)	2.38	0.23	1.77	0.69	4.52
	G/employee	34 (69.4)	15 (30.6)	2.27	0.03	3.37	1.13	10.00
	P/ employee	7 (63.6)	4 (36.4)	2.94	0.22	2.70	0.56	13.05
Presence of animals in home	No	143(80.8)	34 (19.2)	1		1		
	Yes	122 (60.7)	79 (39.3)	2.72	0.00	3.10	1.83	5.27
Fingernail cleanness	No	179 (69.4)	79 (30.6)	1.12	0.07	1.74	0.97	3.14
	Yes	86 (71.7)	34 (28.3)	1		1		
Sex of child	Male	127 (66.8)	63 (33.2)	0.73	0.13	1.43	0.89	2.31
	Female	138 (73.4)	50 (26.6)	1		1		

Key: G/employee-government employee, P/employee-Private employee

### Risk factors of *Giardia lamblia* infestations

On bivariable logistic regression a child’s sex (COR, *P*-value), (2.13, 0.013), place of child spent (COR, *P*-value), (2.088, 0.024), history of deworming (COR, *P*-value), (2.88, 0.002), shoes waring habit (COR, *P*-value), (6.54, 0.00), and presence of latrine (COR, *P*-value), (0.46, 0.02) were had an association with *Giardia lamblia* infestations. But in multivariable logistic regression child sex (AOR, (CI)), (2.235, (1.115–4.480)), place of child spent (AOR, (CI)), (.463, (0.222–.964)), history of deworming (AOR, (CI)), (2.365, (1.091–5.127)), and shoes waring habit (AOR, (CI)), (9.909, (3.313–29.636)) had an association with *Giardia lamblia* infestations (Table 3).

**Table 3:** A multivariable logistic regression analysis of potential risk factors with *Giardia lamblia* and *Hymenolepis nana* infestations identified using formal ether concentration technique among preschool children in Sekota town, Waghimra zone, Ethiopia, 2019, (total positive for GL, *n* = 55, and total negative for GL, *n* = 323), and (total positive for HN, *n* = 19, and total negative for HN, *n* = 359)

		*Giardia lamblia*		*P*-value	COR	*P*-value	AOR	95%CI	
Variables	Categories	Yes (%)	No (%)					Lower	Upper
Child sex	Male	19 (10)	171 (90)		1		1		
	Female	36 (80.9)	152 (19.1)	0.013	2.13	0.023	2.235	1.115	4.480
Place spent	Indoor	17(23)	57(77)		1		1		
	Outdoor	38(12.5)	266 (87.5)	0.024	2.088	0.040	0.463	0.222	0.964
Deworming intake	Yes	12(7.7)	144(92.3)		1		1		
	No	43(19.4)	179(80.6)	0.002	2.88	0.029	2.365	1.091	5.127
Complete vaccination	Yes	44	281		1		1		
	No	11	42	0.17	1.67	0.534	1.347	0.527	3.448
Shoes waring habit	Sometimes	27	243	0. 14	0.58	0.301	0.671	0.315	1.429
	Not wear	15	12	0.00	6.54	0.000	9.909	3.313	29.636
	Regularly	13	68		1		1		
Presence of latrine	Yes	42(17.9)	193(82.1)		1		1		
	No	13(9.1)	130(90.9)	0.02	0.46	0.291	0.633	0.271	1.478
Presence of animal in home	Yes	33(16.4)	168(83.6)	0.27	1.38	0.563	1.243	0.594	2.601
	No	22(12.4)	155(87.6)		1		1		
Cleanness of child finger	Yes	22(18.3)	98(81.7)		1		1		
	No	33(12.8)	225(87.2)	0.16	0.65	0.845	1.071	0.535	2.144
		*Hymenolepis nana*		*P*-value	COR	AOR	95%CI		*P*-value
		Yes (%)	No (%)				Lower	Upper	
Child sex	Male	15(8.4)	174(91.6)	0.007	5.67	7.51	2.07	27.20	0.002*
	Female	3(1.6)	185(98.4)		1	1			
Deworming intake	Yes	4(2.6)	152(97.4)		1	1			
	No	15(6.8)	207(93.2)	0.077	2.75	3.37	1.05	10.8	0.04*
Shoes waring habit	Sometimes	11(4.1)	259(95.9)	0.736	0.82	0.77	0.23	2.59	0.77
	Not wear	4(14.8)	23(85.2)	0.105	3.35	2.43	0.52	11.46	2.43
	Regularly	4(4.9)	77(95.1)		1	1			
Presence of latrine	Yes	15(6.4)	220(93.6)		1	1			
	No	4(2.8)	139(97.2)	0.13	0.42	0.31	0.09	1.00	0.05

Key: GL- *Giardia lamblia*, and HN- *Hymenolepis nana*, *P-value is less than 0.05

### Risk factors of *Hymenolepis nana* infestations

On bivariable logistic regression a child’s sex (COR, *P*-value), (5.67, 0.007), and presence of latrine (COR, *P*-value), (0.42, 0.13) were had an association with *Hymenolepis nana*. But in multivariable logistic regression child sex (AOR, (CI)), (7.51, (2.07–27.20)), and history of deworming (AOR, (CI)), (3.37, (1.05 -10.8)), were had an association with *Hymenolepis nana* (Table 3).

## Discussion

The prevalence of intestinal parasitic infestations among preschool children in Sekota town was 83 (21.9%) with 95% CI of (17.7–26.3%), and 113 (29.9%) with 95% CI of (25.1–34.8%) on wet mount and formal ether concentration techniques respectively. A total of five species of intestinal parasites were identified on wet mount. But on formal ether concentration technique only 4 parasites were identified with the highest prevalence of *Giardia lamblia* cys*t* 55(14.6%), *Hymenolepis nana* 19(5%), *Entamoeba histolytica\dyspar* cyst 17(4.5%), and *Ascaris lumbricoides* 6(1.6%), as well as 6 (1.6%) were to be mixed infestations. The prevalence of hook worm in wet mount was 6 (1.6%) but in formal ether concentration there was no positive results identified.

The prevalence of IP in this study agreed with a study done in Uganda that reported the prevalence of IP infestation was 32.8% [22]. But the finding of this study is higher than a study done in Tanzania that reported the prevalence was 15.1% [23]. The discrepancy might be because of long period of study difference and improvement of health care services and socio-demographic status over time.

The present study had lower prevalence of IP in relative to other studies; such as a research done in Eritrea that reported 46 (36.50%) [24], in Sudan 64.4% [25], and in southern sudan 44% [26]. The difference might be because of different study population. The study population in this study is children aged 2 to 6 years but all the referred studies studied on school aged children at primary schools.

The finding of this study was higher than a studies, that were reported from researches done at Dessie referral hospitals was 15.5% among children below 5 years old [27], at Debre-Berhan, Ethiopia among children below 5 years old was 17.3% [28], at University of Gondar Hospital, Gondar, Northwest Ethiopia was 48 (17.3%) among children below 5 years old [29], and at Wonji Shoa, Sugar Estate was 24% [30].

The current study had a lower prevalence of IP than a study done in Shesha-Kekele, Wondo Genet, Southern Ethiopia, the prevalence of IP was 85.1% among under five children. The prevalence of *T. trichiura, S. mansoni, Ascaris lumbricoides, Hymenolepis nana,* and *hookworm* infestations in Wondo Genet were 74.7, 37.2, 25.7, 4.5, and 5.9%, respectively [31].

This high difference might be the result of geographic variation. In which, Shesha-Kekele is known for having high prevalence of Soil transmitted helminthiasis, and intestinal schistosomiasis [32], and the time period has also a significant impact since the referred study was done before 8 years of the current study. Many things like quality of health care, socio-economic status, access to clean water, and improved sanitation as well as awareness of the community, particularly, the mother’s awareness was improving over time. The finding of the study is also lower than the study done in Jimma that was 41.1% [33]. The difference might be due to stool sample examination techniques used.

This study is in line with a study done in Gonder that reported the overall prevalence as 34.2% among primary school students [34]. The study is also in agreement with a study done in Hawasa that reported the prevalence as 26.6% [35]. This similarity might be due to similar study setting, in which all studies are done in an urban setting.

The finding of this study was also lower than a study done in Bahir Dar (65.5%) [36], Dagi primary school (77.9%) [37], and Motta Town (68.4%) [38]. This variation might be the result of different study periods, which have done before 2, 6 and 5 years respectively. This time difference affects the quality of health care service, when the time is late the care is also coming better and better. There is also a difference in the study population and setting. Those three studies done on school aged children comes from either Urban or rural but this study done on urban preschool children.

The present study stated *Giardia lamblia cyst* 55(14.6%), *Hymenolepis nana* 19(5%), and *Entamoeba histolytica\dyspar cyst* 17(4.5%) as the top 3 species respectively. But the study from Gonder University hospital reported the predominant 3 parasites were *Ascaris lumbricoides* 19 (35.8%), *Hymenolepis nana* 13 (24.5%) and cyst of *Giardia lamblia* 5 (9.4%) [29]. A study in Gonder community school also indicated the predominant intestinal parasites detected were *Entamoeba histolytica/dispar,* followed by *Hymenolepis nana* and *Ascaris lumbricoides* [34].

The most predominant parasite identified in this study is *Giardia lamblia cyst* 55(14.6%). This is agreed with some studies that indicated the predominant parasite among children was *G. lamblia* [25, 28, 33, 39, 40]. But, in some other studies the most encountered parasite was *E. histolytica* [29, 34, 35].

In this study 18 (4.8%) of children were had double parasitic infections whereas studies conducted in Debre Birhan, and Gonder university Hospital reported only five double infections [28, 29], and in Hawassa six children were infected with two parasites [35] but a study in Bahir Dar reported 58 (16.2%) double infections [36].

Children who had no history of deworming increases the odds of intestinal parasitic infestation 2.3 times (AOR, 95%CI), (2.32, 1.32–4.09) than their counterparts, who have a history of deworming. The finding is supported with the reports of WHO, and World Bank (WB), that states deworming decreases intestinal parasitic infestations [41, 42].

Children who lived in a single room with domestic animals have high odds of infestation with intestinal parasites 3.1 times (AOR, (95% CI) (3.10, (1.83–5.27) than their counterparts, which is in line with a study done in Taiwan, (AOR, 4.23, 95% CI, 1.10–16.39), and *P* value of 0.036) [43], and Burkina Faso (*P* = 0.008) that reported as animals increase the odds of intestinal parasitic infestations [44]. This might be either animals transmit zoonotically disease directly or cause to compromise the cleanness of the home indirectly. The lack of cleanness is a risk factor for intestinal parasitic infestations.

Children born and raised from governmental employees had high odds of intestinal parasitic infestations 3.4 times (AOR, (95% CI) (3.37 (1.13–10.00) than children of merchants. This might be due to the inflated shortage of water in Sekota town. Therefore, those civic servants might not able to fetch sufficient water regularly because of time constraint and this might contribute for intestinal parasitic infestation in addition to shortage of time to care their children than small scale merchants, who spent in indoor with their children by doing their job like shopping, or restaurant or bar.

Modified acid-fast staining technique was not used to detect Cryptosporidium species, and it might cause to miss this species. As the collection period was short, potential seasonal fluctuations might have affected the actual prevalence.

The association run for *Hymenolepis nana* and *Giardia lamblia* less reliable as the power of the sample for these parasites is weak. But this might be used as a baseline for further investigation of the factors using large sample size.

## Conclusion

The prevalence of intestinal parasitic infestations in Sekota town is high, which is a public health problem. The parasites identified in the town were Entamoeba histolytica\dyspar cyst, *Giardia lamblia* cyst, Ascaris lumbricoides, Hookworm on wet mount only, and Hymenolepis nana. The risk factors that contribute to intestinal parasitic infestations in this study were preventable and modifiable. Those factors were, not using medication for deworming, having animals in the same living room, and being governmental employees as an occupation.

Therefore, the concerned bodies need to emphasis on periodical deworming, and separating living rooms from animals. In addition, campaign-based health education or frequent visit to the houses in the town may decrease the prevalence of Intestinal infestations.

## Data Availability

The raw materials that support the conclusions of this research will be available to researchers, who need the data to use for non-commercial purposes through requesting the authors.

## Abbreviations

AOR: Adjusted odds ratio
CI: Confidence interval
IP: Intestinal parasites
SOP: Standard operational procedure
SPSS: Statistical package for social science
WB: World Bank
WHO: World Health Organization
